# HIPK2 in Colon Cancer: A Potential Biomarker for Tumor Progression and Response to Therapies

**DOI:** 10.3390/ijms25147678

**Published:** 2024-07-12

**Authors:** Alessandra Verdina, Alessia Garufi, Valerio D’Orazi, Gabriella D’Orazi

**Affiliations:** 1 Unit of Cellular Networks and Molecular Therapeutic Targets, IRCCS Regina Elena National Cancer Institute, 00144 Rome, Italy; alessandra.verdina@ifo.it (A.V.); alessia.garufi@ifo.it (A.G.); 2 Department of Surgery, Sapienza University, 00185 Rome, Italy; valerio.dorazi@uniroma1.it; 3 Department of Neurosciences, Imaging and Clinical Sciences, University “G. D’Annunzio”, 66013 Chieti, Italy

**Keywords:** HIPK2, p53, colorectal cancer, colon cancer, hypoxia, hyperglycemia, cancer therapy, Wnt/β-catenin, NRF2, microRNA, angiogenesis, tumor inflammation

## Abstract

Colon cancer, one of the most common and fatal cancers worldwide, is characterized by stepwise accumulation of specific genetic alterations in tumor suppressor genes or oncogenes, leading to tumor growth and metastasis. HIPK2 (homeodomain-interacting protein kinase 2) is a serine/threonine protein kinase and a “bona fide” oncosuppressor protein. Its activation inhibits tumor growth mainly by promoting apoptosis, while its inactivation increases tumorigenicity and resistance to therapies of many different cancer types, including colon cancer. HIPK2 interacts with many molecular pathways by means of its kinase activity or transcriptional co-repressor function modulating cell growth and apoptosis, invasion, angiogenesis, inflammation and hypoxia. HIPK2 has been shown to participate in several molecular pathways involved in colon cancer including p53, Wnt/β-catenin and the newly identified nuclear factor erythroid 2 (NF-E2) p45-related factor 2 (NRF2). HIPK2 also plays a role in tumor–host interaction in the tumor microenvironment (TME) by inducing angiogenesis and cancer-associated fibroblast (CAF) differentiation. The aim of this review is to assess the role of HIPK2 in colon cancer and the underlying molecular pathways for a better understanding of its involvement in colon cancer carcinogenesis and response to therapies, which will likely pave the way for novel colon cancer therapies.

## 1. Introduction

**Figure 1:**
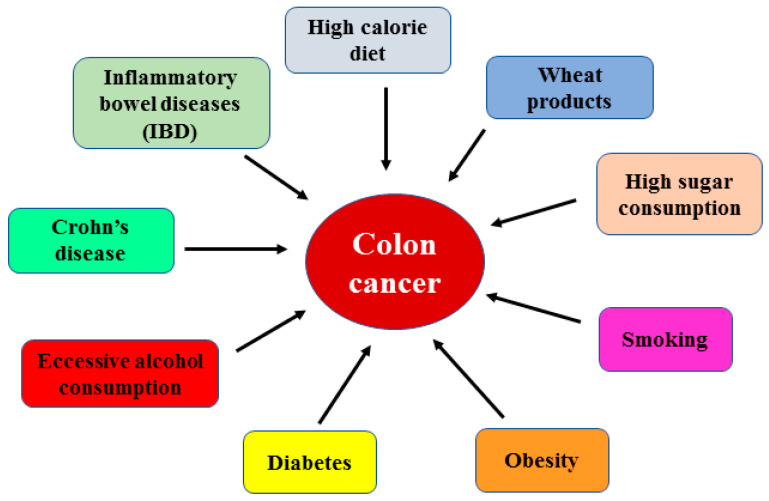
Schematic representation of the risk factors of colon cancer development.

Colorectal cancer (herein, colon cancer), which comprises colon and/or rectum cancer, is one of the most common cancers in women and men: it is the third most frequently diagnosed type of cancer (11% of all diagnosed cancer cases) and the second leading cause of cancer-related mortality worldwide [1,2]. The 5-year survival rate depends on the tumor stage, ranging from 90% for the localized stage to 71% for regional and 14% for patients with distant metastasis [3]. Thus, early detection of the disease is a priority. In addition, a worrying rise in patients presenting with colon cancer younger than 50 years has been observed [4]. There are three principal types of colon cancer: sporadic, hereditary and colitis-associated. Global incidence and mortality are likely to be increased in the coming decades and especially in highly developed countries, mostly depending on dietary factors and lifestyle, family history and chronic inflammation [5,6]. Risk factors comprise a high-calorie diet rich in animal-derived proteins, especially red and processed meat, wheat products and high sugar consumption, combined with poor physical activity. In addition, other risk factors are family history of colon cancer (stronger association for first-degree relatives), inflammatory bowel disease (IBD), Crohn’s disease, smoking, excessive alcohol consumption, obesity and diabetes [7,8] (Figure 1).

**Figure 2:**
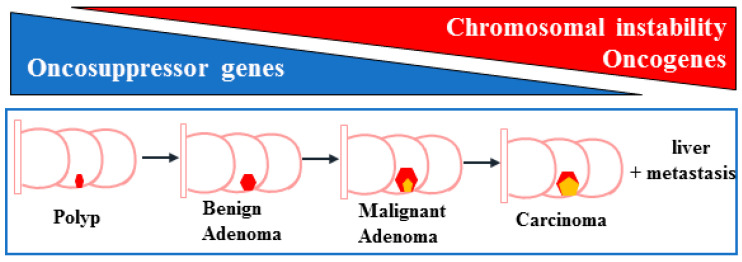
Schematic representation of the steps of colon cancer development. The passages from polyp to benign adenoma, malignant adenoma, carcinoma (represented by the pink/yellow pentagons), in the colon regions, and liver metastasis, include the reduction of oncosuppressor genes (blue triangle) and the activation of oncogenes with increased chromosomal instability (red triangle).

The most important preventive factor represents routine endoscopic check-ups, namely colonoscopy, with the removal of precancerous lesions (polypectomy) and, interestingly, the role of new technologies in detection of neoplasia, such as artificial intelligence, which is rapidly emerging [9]. Colon cancer development is characterized by a “multistep carcinogenesis“ process, according to the Fearon and Vogelstein genetic model [10], involving a series of histological and morphological changes beginning as a benign adenomatous intestinal polyp from epithelial tissue of the colon, which progresses to advanced adenoma with high-grade dysplasia, invasive adenocarcinoma, and metastasis to distant organs such as the liver [10] (Figure 2). These changes are triggered by a sequential accumulation of specific genomic alterations involving different oncogenes such as Kirsten rat sarcoma virus (KRAS), serine/threonine-protein kinase B-Raf (BRAF), phosphatidylinositol kinase catalytic subunit alpha (PIK3CA) and tumor suppressor genes such adenomatous polyposis coli (APC), tumor suppressor protein (TP)53, SMAD4, phosphatase and tensin homolog (PTEN) [11,12], along with chromosomal (microsatellite) instabilities (Figure 2), that deregulate key signaling pathways driving disease progression, including Wnt/β-catenin, downstream mitogen-activated protein kinase (MAPK), phosphoinositide 3-kinase (PI3K), epidermal growth factor receptor (EGFR) and transforming growth factor beta (TGF-β) [13]. In the early steps of tumorigenesis, the function of the tumor suppressor p53 is inhibited by increased levels of its suppressor MDM2 (murine double minute 2) caused by a *KRAS* mutation actioned by the PI3K–AKT pathway [14,15]. Later stages of tumorigenesis are induced by a further loss of wild type p53 protein, by gene mutation, leading to a more malignant phenotype and resistance to therapies [16,17,18].

Recently, the impact of the tumor microenvironment (TME)—composed of cancer-associated fibroblasts (CAF), endothelial cells sprouting angiogenesis, immune cells, along with the microbiota and the tumor-derived exosomes (TEXs)—has gained attention in colon cancer progression and metastasis, either by promoting or inhibiting the processes, becoming an interesting target in colon cancer therapies [19,20]. The basis of colon cancer treatment consists of surgery, targeted therapy, neoadjuvant radiotherapy and adjuvant chemotherapy along with the promising immunotherapy [21,22]. Unfortunately, drug-resistance remains one of the deadlocks for the low survival rates of colon cancer patients, as several molecular alterations confer resistance to standard chemotherapy and targeted agents [21]. A better understanding of the mechanisms leading to intrinsic and acquired resistance to therapies will be a great asset for prognostic and therapeutic purpose in colon cancer. Colon cancers carrying common genetic events have been shown to markedly vary in their biology [23]; thus, to further improve treatment opportunities, additional diagnostic parameters and molecular determinants of clinical outcome for colon cancer are required.

In this regard, the aim of this review is to assess the role of the “bona fide” oncosuppressor homeodomain interacting protein kinase-2 (HIPK2) in colon cancer progression and response to therapies. By reviewing the current literature, the review will summarize the molecular interplay that occurs between HIPK2 and molecular pathways involved in colon cancer regression and response to therapies that could likely be exploited as a potential biomarker and therapeutic target in colon cancer therapy.

## 2. HIPK2 in Colon Cancer

HIPK2 is a serine-threonine and an evolutionarily conserved protein kinase that interacts with many different molecules by means of its kinase or transcriptional activities [24,25]. Through these interactions, HIPK2 regulates many cellular functions including cell growth and apoptosis, fibrosis, angiogenesis and inflammation involved in cancer, fibrotic and neurodegenerative diseases [26,27,28]. Scientific consensus suggests that HIPK2 is a “bona fide” oncosuppressor protein, thus, its activation inhibits tumor growth, mainly by induction of apoptosis with p53 activation, while its inactivation increases tumorigenicity and chemoresistance through inhibition of apoptotic pathways or de-repression of pro-survival pathways [29]. The role of HIPK2 in cancer has been previously well reviewed [24,25,26,27,28,29]. Briefly, HIPK2 is activated by anticancer drugs or DNA damage and inactivated by hypoxia, hyperglycemia or micro (mi) RNAs [29,30,31]. The role of HIPK2 in colon cancer comes from studies that demonstrate that HIPK2 participates in most of the pathways affected in colon cancer: it positively modulates oncosuppressor p53 through direct phosphorylation, inducing apoptosis in response to DNA damage [32,33]; it interacts with the Wnt/β-catenin and TGF-β at multiple levels (e.g., by targeting β-catenin, by forming a multimeric complex with AXIN, as a transcriptional corepressor of SMADs and by interacting with the common Wnt/TGF-β mediator TAK1) [34,35,36,37,38,39,40,41]; and it interacts with the nuclear factor erythroid 2 (NF-E2) p45-related factor 2 (NRF2) [42,43,44]. Recently, a role for HIPK2 in the remodeling of the TME has been unveiled. In particular, HIPK2 inhibition induces a pro-inflammatory phenotype, angiogenesis and cancer-associated fibroblasts (CAF) differentiation [27,45,46,47], supporting tumor progression and chemoresistance. HIPK2 is downregulated by hypoxia and by hyperglycemia [48,49] (the latter being a common feature of diabetes mellitus), two conditions often present in solid tumors, including colon cancer, and having a role in cancer progression and resistance to therapies [5,50,51]. For these reasons, HIPK2 is becoming an appealing molecule in the colon cancer field, deserving in-depth analysis in future studies, for further understanding of colon cancer development and response to therapies. Below is a summary of the studies that, up to now, have evaluated the HIPK2 expression in colon cancer tissues, genomic data sets and cell lines, with a focus on the interaction with molecular pathways involved in cancer progression and response to therapies.

### 2.1. HIPK2 and p53

The TP53 protein is considered “the guardian of the genome”, as it plays a crucial role in regulation of the cell cycle and the genome stability [52]. Given its key role in drug-induced apoptosis, wild-type (WT) p53 plays a critical role in efficient tumor response to therapies [53]. Up to 60% of patients with colon cancer show somatic mutations of the *TP53* gene and in the remaining percentage, p53 can be deregulated at the protein level. In both cases, inactivation of p53 oncosuppressor functions drives the transition from adenoma to adenocarcinoma and impairs the tumor response to therapies [14,16,18]. As HIPK2 is a positive regulator of p53 apoptotic function [54], the effect of HIPK2 depletion on p53 transcriptional activity was first analyzed using a microarray assay. The study highlighted that HIPK2 depletion with small interfering (si) RNA in colon cancer cells leads to the loss of WTp53 target gene activation [55]. Mechanistically, the results showed that stable HIPK2 knockdown, by modulating metallothionein and zinc, induces WTp53 protein misfolding that impairs p53–DNA binding and WTp53 target gene transcription. Interestingly, the WTp53 misfolding could be reverted by zinc supplementation, as evidenced by in vitro and in vivo studies [55,56,57]. Bioinformatics analysis of microarray data from colon cancer patients with known clinical record and p53 mutation status, using the Kaplan–Meier procedure, showed significant association of poor survival with low HIPK2 expression only in tumors expressing WTp53, in agreement with the in vitro data [55] (Table 1). These findings unveiled an unexpected mechanism of WTp53 inactivation following HIPK2 depletion and proposed a new way (that is, zinc supplementation) to modify the possible equilibrium between active and inactive WTp53 in tumors (i.e., by increasing WTp53 oncosuppressor function), which may be applied in the clinic. These findings were in line with a study showing that *HIPK2* mutations in acute myeloid leukemia impair p53-mediated transcription [58]. HIPK2 protein activity was also required in vivo for efficient p300/p53 co-recruitment onto apoptotic gene promoters; thus, by balancing p53 acetylation and deacetylation [59], other than through phosphorylating p53 at serine 46 (Ser46) [53,54], HIPK2 regulates p53 apoptosis-promoting transcriptional activity [54]. These pioneering studies revealed a critical role of HIPK2 in maintaining the transactivation activity of WTp53 and further suggest that low expression of HIPK2 may impair the p53 function in tumors harboring WTp53. They also opened the way to anticancer treatments using zinc, in combination with classical chemotherapy, to restore WTp53 activity when cancers present low expression of HIPK2 [57] or in p53-functionally deficient cancer cells [60].

Looking for a prognostic role of HIPK2 in colon cancer, the authors analyzed a retrospective series of 80 primary patients’ samples at different tumor stage of colon cancer and normal mucosa samples (taken at distance from the tumor) by immunofluorescence and tissue microarray (TMA) by using multiplexed tissue cytometry capable of exploring correlative protein expression at the single tumor cell level on TMA, simultaneously monitoring HIPK2 and p53 protein expression at the single cell level. The authors found that an increase of HIPK2 protein level in the tumor, compared to the normal mucosa, in univariate analysis, was associated with a better prognosis that did not depend on WTp53 status because it was also observed in p53-mutated background, highlighting the p53-independent function of HIPK2 [61] (Table 1). The expression of HIPK2 was then evaluated in primary tumor specimens of human colon cancer, with particular regard to post-operative cancer recurrence, metastasis and malignancy grades [62]. The authors started from the finding that in China, the rate of colon cancer incidence is increasing faster nationally than all other cancers [63] and that colon tumorigenesis may depend on functional abnormalities of relevant genes. Immunohistochemistry (IHC) analysis of 100 colon tumor samples and 20 normal intestinal tissues displayed that HIPK2 expression inversely correlates with Dukes stage and depth of cancer invasion [62] (Table 1). This is in line with previous studies showing that HIPK2 protein downregulation in samples of other cancer types, such as breast [64], thyroid [65], and pancreatic [66], increases tumor progression. By using a xenograft colon cancer mouse model, the authors tested the in vivo anti-tumor effect of verbascoside (VB), an active constituent of a Chinese traditional medical plant genus that has an effect in many cancers, including colon cancer [67,68], and measured protein levels of HIPK2 and p53, and the apoptosis-related gene products Bax and Bcl-2. They showed that VB stimulates the HIPK2–p53 signaling pathway, thus inhibiting cell proliferation and promoting apoptosis in colon cancer [62] (Table 1), pointing again to the key role of a functional HIPK2–p53 axis for achieving efficient tumor cell death in response to cytotoxic therapies [26].

**Table 1:** Summary of the HIPK2 expression in colon cancer tissues and cell lines along with the biological and molecular effects and the specific references. IHC: Immunohistochemistry; cPLA2: cytosolic phospholipase A2; PGE2: prostaglandin E2; COX-2: cyclooxygenase-2; VEGF: vascular endothelial growth factor; HIF-1: hypoxia inducible factor-1; TMA: tissue microarray; 5-FU: 5-Fluorouracil; OXA: oxaliplatin; lncRNA PRNT: long non-coding RNA prion protein testis specific.

HIPK2 Expression in Colon Cancer Tissues and/or Cell Lines	Molecular/Cellular Effects	Biological Outcome	Ref.
↓ HIPK2 protein expression by siRNA in colon cancer cell lines in vitro; Microarray data on >300 samples from colon cancer patients with known clinical records and p53 mutation status.	↓ p53 apoptotic activity in vitro and in vivo in tumor xenografts	↓ Patient survival with low HIPK2 expression only in tumors expressing WTp53	[55]
↑ HIPK2 protein levels, by multiplexed tissue cytometry, in 80 colon cancer tissues		↑ Patient prognosis	[61]
HIPK2 protein expression by IHC in 100 colon cancer tissues	Verbascoside treatment stimulates HIPK2–p53 apoptotic pathway in vitro and in vivo, in tumor xenografts	Inverse correlation between HIPK2 expression and Dukes stage and invasion	[62]
↓ HIPK2 mRNA levels in nine colon cancer tissues of patients with sporadic colorectal cancer	↑ cPLA2 mRNA expression and ↑ PGE2 production; HIPK2 represses cPLA2 promoter activity in vitro.	↓ HIPK2 expression increases the growth of tumor xenografts; Inverse correlation between HIPK2 expression and Dukes stage.	[45]
Inverse correlation between HIPK2 and COX-2 expression in primary colon adenocarcinomas in silico	↓ HIPK2 expression leads to ↑ COX-2/VEGF pathway through HIF-1 in vitro; VEGF inhibition in HIPK2-depleted cells restored dendritic cell (DC) maturation.		[69]
Colitis-associated colon cancer in *HIPK2+/−* mice	↑ Percentage of macrophages in the tumors of *HIPK2+/−* mice; ↑ Serum concentration of pro-inflammatory IL-6, IL-1β and TNF-α cytokines through NF-κB; HIPK2 inhibits NF-κB activity in vitro.	↑ Colon cancers grow in *HIPK2+/−* mice	[46]
↑ HIPK2 expression, by IHC, in TMA of 270 colon cancer samples	↑ Tumor progression and TNM stages; ↓ HIPK2 expression reduces ERK phosphorylation in vitro.	↓ HIPK2 expression reduces the growth of tumors derived from KRAS mutated colon cancer cells	[70]
↑ HIPK2 expression, by immunohistochemistry (IHC), in tissue microarray (TMA) of 84 stage II colon cancer samples		↑ Response to therapy (5-FU, OXA)	[71]
↑ lncRNA PRNT in datasets of OXA-resistant cancer cells	↓ HIPK2 mRNA	↑ Colon cancer cell growth and migration, and resistance to OXA in vitro and in vivo	[72]
↓ HIPK2 mRNA levels in colon cancer tissues	↑ exomiR-1229, ↑ VEGFA, ↑ VEGFR1, and ↑ p-AKT; exomiR-1229 target and inhibits HIPK2 in vitro.	↑ Angiogenesis, metastasis and poor survival	[73]
**HIPK2 Expression in Colon Cancer Tissues and/or Cell Lines**	**Molecular and Biological Effects**		**Ref.**
Reduced HIPK2 protein expression by siRNA in cell lines	Reduced p53 apoptotic activity in vitro and in vivo; Poor patient survival with low HIPK2 expression only in tumors expressing WTp53.		[55]
Increased HIPK2 levels in CRC tissues	Better patient prognosis that did not depend on WTp53 status		[61]
HIPK2 expression by IHC in CRC	Inverse correlation between HIPK2 expression and Dukes stage and invasion; Verbascoside treatment stimulates HIPK2/p53 apoptotic pathway in vitro and in vivo.		[62]
Low HIPK2 mRNA levels in CRC tissues of patients with sporadic colorectal cancer	High cytosolic phospholipase A2 (cPLA2) expression and PGE2 production; HIPK2 represses cPLA2 promoter activity; Increased in vivo tumor growth of xenografts from HIPK2-depleted colon cancer cells; Inverse correlation between HIPK2 expression and Dukes stage.		[45]
Inverse correlation between HIPK2 and COX-2 expression in primary colon adenocarcinomas in silico	HIPK2 inhibition leads to upregulation of COX-2/VEGF pathway through HIF-1; VEGF inhibition in HIPK2-depleted cells restored dendritic cell (DC) maturation.		[69]
Colitis-associated CRC in *Hipk2+/−* mice	Tumors grow more rapidly in *Hipk2+/−* mice; The percentage of macrophages is increased in the tumors of *Hipk2+/−* mice; Increased serum concentration of pro-inflammatory IL-6, IL-1β and TNF-α cytokines through NF-κB; HIPK2 inhibits NF-κB activity.		[46]
Increase of HIPK2-positive cancer cells in TMAs of colon cancer samples	Correlation with tumor progression and TNM stages; HIPK2 knockdown reduces ERK phosphorylation in vitro and the growth of tumors derived from KRAS mutated cells.		[70]
High HIPK2 positivity in TMAs from tumor samples	Association with improved response to therapy (5-FU, OXA), independent from p53 status		[71]
Inverse correlation between HIPK2 mRNA and lncRNA PRNT in datasets of OXA-resistant cancer cells	PRNT regulates HIPK2 expression in CRC by sponging ZNF184 transcription factor		[72]
HIPK2 mRNA downregulation in CRC tissues compared to the adjacent normal tissues	HIPK2 inhibition by exomiR-1229 with consequent angiogenesis by VEGFA, VEGFR1 and p-AKT upregulation		[73]

↑ high; ↓ low.

### 2.2. HIPK2 and Inflammatory Pathways (COX/PGE2/VEGF and NF-κB) 

HIPK2 mRNA levels were found to be significantly lower in colon cancer tissues of patients with sporadic colorectal cancer, expressing cytosolic phospholipase A2 (cPLA2), compared to tissues of patients with familial adenomatous polyposis, which showed undetectable cPLA2 levels [45]. An interesting link between HIPK2 and cPLA has been revealed. cPLA2 is involved in the generation of inflammatory prostanoids, including prostaglandin E2 (PGE2), which is the predominant prostanoid found in most colon cancers and is known to promote colon carcinoma growth by stimulating proliferation, angiogenesis, invasiveness and by inhibiting apoptosis [74,75]. In vitro studies with colon cancer cell lines, using siRNA, showed that HIPK2 silencing is associated with increased PGE2 biosynthesis that was profoundly suppressed by the cPLA2 inhibitor. Thus, molecular studies showed that HIPK2 overexpression, in a complex with histone deacetylase 1 (HDAC1), inhibits the cPLA2-luciferase promoter activity [45] (Table 1). The tumors derived from HIPK2-silenced cells, injected in nude mice, showed noticeably increased growth compared with tumors derived from parental cells, underscoring the role of HIPK2 to restrain progression of human colon tumorigenesis, at least in part, by turning off cPLA2-dependent PGE2 generation [45]. As a proof of principle, analysis of HIPK2 mRNA expression in human colon cancers showed that it tended to correlate inversely with the Dukes staging of the tumors [45] (Table 1), in agreement with the Zhou and colleagues’ study [62].

The production of prostaglandins via ciclooxygenase-2 (COX-2) is an important signaling pathway in the pathogenesis of colon cancer [76,77]. Transcriptional control of the COX-2 gene may depend on Wnt/β-catenin [78] or hypoxia-inducible factor-1 (HIF-1) [79] signaling pathways that can both undergo activation when HIPK2 is inhibited [29]. Thus, HIPK2 knockdown, by using siRNA, in colon cancer cells has been shown to upregulate COX-2 expression and, by using the Oncomine integrated cancer database research tool (http://www.oncomine.org, accessed on 23 June 2024), the analysis of datasets, obtained from specimens of normal tissues and primary colon adenocarcinomas, revealed an inverse correlation between *HIPK2* and *COX-2* expression [69] (Table 1). Activation of COX-2 and HIF-1 pathways can induce vascular endothelial growth factor (VEGF) expression [80,81], which in turn potentiates HIF-1 transcriptional activity and contributes to tumor progression [82]. Inflammatory mediators such as VEGF have been shown to suppress dendritic cell (DC) maturation, permitting evasion of anti-tumor immune responses, and to induce tumor progression [83,84]. In this regard, HIPK2 knockdown, by using siRNA, has been shown to generate a proinflammatory phenotype by upregulating COX-2/VEGF pathway through HIF-1 [85], then, VEGF inhibition in HIPK2-depleted cell culture, by the use of anti-VEGF monoclonal antibody, efficiently restored DC maturation [69] (Table 1). Therefore, colon cancer progression and resistance to therapies may worsen, not only in the presence of chronic inflammatory diseases such as inflammatory bowel disease (IBD) [86], obesity [87] or hyperglycemia [51], but also by an inflammatory phenotype induced by genetic or epigenetic changes of HIPK2.

Inflammation is a hallmark of cancer and may arise from a variety of factors including infections, environmental carcinogens (e.g., smoking, alcohol, radiation), cellular senescence and obesity [83]. Chronic inflammation is an important driver to the development of many cancers, such as colon cancer and hepatocellular carcinoma (HCC) [88]. Starting from the findings that the tumor suppressor gene HIPK2 was expressed at higher levels from IL-6low HCC samples than from IL-6high HCC samples, based on the cluster analysis of protein kinases in hepatitis B virus infection-associated HCC patient samples, the authors analyzed the potential regulatory function of HIPK2 in chronic inflammation-driven cancers by generating *HIPK2* knockout (KO) mice [46]. Because *HIPK2* is known to be haploinsufficient [36,89], the authors mainly used *HIPK2+/−* mice that grow normally as wild-type (WT) mice. MC38 colon carcinoma cells were subcutaneously injected into WT and *HIPK2+/−* mice, and the authors found that tumors grow more rapidly in *HIPK2+/−* mice and that, surprisingly, the percentage of macrophages is increased in the tumors of *HIPK2+/−* mice [46]. In vivo studies demonstrated that *HIPK2*-deficient mice are more susceptible to lipopolysaccharide (LPS)-induced endotoxemia and cecal ligation and puncture (CLP)-induced sepsis, with increased serum concentration of proinflammatory IL-6, IL-1β and TNF-α cytokines [46]. In vitro studies showed that *HIPK2* knockdown significantly enhances the messenger RNA (mRNA) levels of *Il6*, *Il1b* and *Tnf-α*, as well as the IL-6 and TNF-α protein levels [46]. Mechanistically, the authors found that, in macrophages, HIPK2 phosphorylates histone deacetylase 3 (HDAC3) at serine 374 to inhibit its deacetylase activity, thus suppressing NF-κB/p65 activation and the production of proinflammatory cytokines [46]. Induction of NF-κB-mediated transactivation is critical to induce inflammation, therefore, unveiling novel mechanisms that specifically regulate NF-κB activation will provide important therapeutic applications in inflammatory diseases. In this regard, the findings of Zhang and colleagues propose to target NF-κB through the axis HIPK2–HDAC3–NF-κB to ameliorate not only colitis-associated colorectal cancer and sepsis but also inflammation-related cancers [46] (Table 1). These findings are in line with previous ones showing that overexpression of HIPK2 suppresses the expression levels of proinflammatory TNF-α and IL-1β proinflammatory cytokines in spinal cord-injured rats [90] and attenuates sepsis-mediated liver injury [91] and that, in cancer cells, HIPK2 can block the inflammatory phenotype induced by VEGF/PGE2 production [45,69], underlining an important role for HIPK2 in suppressing inflammation-induced tumor progression.

### 2.3. HIPK2 and Liver Metastasis 

Liver metastasis of colon cancer is an important cause of death, but its molecular mechanisms are still unclear [92]. In a pilot study aiming at investigating new markers and therapeutic targets of colon cancer liver metastasis, the authors performed high-depth whole-exome sequencing of primary tumor tissues, matched paracancerous, normal tissues, and liver metastases of four colorectal cancer patients. They found that the genes with the highest mutation frequency were *titin* (TTN), *obscurin* (OBSCN), and *HIPK2* [93]. The very limited number of patient samples prevented demonstration of any significant involvement of those genes in colon cancer development and metastasis; therefore, additional studies are necessary to correlate the potential high frequency of mutations in the *HIPK2* gene with colon cancer development and liver metastasis.

### 2.4. HIPK2 and Mutant KRAS

Opposite to the above findings, a role for HIPK2 as positive mediator of colon cancer progression has been proposed. Through the production of TMAs of cancer samples from a retrospective series of 270 stage I to IV patients with colon cancer, the HIPK2 protein expression was evaluated by IHC analyses. The authors found that the number of HIPK2-positive cancer cells increases with tumor progression and correlates with the TNM stages. Results from next generation sequencing (NGS) analysis revealed that high HIPK2 expression significantly associates with risk of colon cancer recurrence [70]. By using overexpression or silencing experiments with the CRISPR/Cas9 technology, the authors found that HIPK2 expression increases when KRAS pathway is activated and that in HIPK2-knockout cells the phosphorylation of extracellular signal regulated kinase (ERK) and its upstream activator, MEK, in basal condition and after KRAS pathway activation, is impaired compared with the HIPK2-WT cells [70]. In agreement, HIPK2 silencing reduced the growth of tumors derived from KRAS mutated cells [70] (Table 1). These data counteract the previous data showing that HIPK2 depletion increases colon cancer growth compared to control tumors, depending on activation of several tumor-promoting pathways, and inversely correlates with the stage of colon cancer [37,45,62]. They also counteract the finding that HIPK2 overexpression reduces ERK phosphorylation and pancreatic cancer proliferation, making HIPK2 a suitable target for inhibiting KRAS/ERK activation in pancreatic cancer [66]. Therefore, further studies are necessary to demonstrate the pro-tumorigenic role of HIPK2 in the context of mutant KRAS and the mechanisms that led to increased HIPK2 protein levels, as the authors did not define it [70]. In this regard, it is known that oncogenic KRAS induces nuclear factor erythroid 2 (NF-E2) p45-related factor 2 (NRF2) expression, conferring chemoresistance [94], and that NRF2 impairs the HIPK2 apoptotic activity [42] (see below). Therefore, it could be interesting to assess whether, in the cooperation between mutant KRAS and HIPK2, NRF2 might have a role and whether the HIPK2 pro-survival role may depend on posttranslational modifications [95,96] leading to HIPK2 protein stability unbalancing its pro-survival/apoptotic functions. Thus, previous findings support the existence of a “HIPK2 protein modification code” [97,98] that might fine-tune HIPK2 as a signaling hub to determine cell fate, depending on cellular context.

### 2.5. HIPK2 and Response to Colon Cancer Chemotherapy

Using part of the above TMA of primary human colon tumor samples [70], the authors analyzed 84 patients with stage II colon cancer, treated or not with adjuvant chemotherapy, to evaluate the contribution of HIPK2 in chemotherapy response [71]. Stage II colon cancer accounts for ~25% all colon cancer cases and it is an early-stage tumor without lymph node spread or distant sites, and with low risk of recurrence. However, the management of stage II colon cancer after surgical resection remains a clinical dilemma due to the lack of reliable criteria for identifying patients who may benefit from adjuvant chemotherapy [99]. The authors showed that TMAs from normal colon tissues exhibit a low level (≤5%) of HIPK2-positive cells. By contrast, TMAs from tumor samples exhibit a broad range of HIPK2 positivity with up to 50% positivity that associated with an improved response to therapy which was independent from p53 status [71] (Table 1). In vitro studies using the CRISPR/Cas9 technology confirmed that, compared with Ctrl-Cas9 cells, HIPK2-knockout significantly decreases cell sensitivity to 5-Fluorouracil (5-FU) and oxaliplatin (OXA) [71], drugs frequently used in the treatment of colon cancer, including stage II cases [100]. Therefore, the authors concluded that HIPK2 protein expression is associated with chemo-response in early-stage colon cancer, which represents an initial step toward defying a novel predictive marker for patients with stage II colon cancer who may benefit from adjuvant therapy [71]. In a recent study trying to identify long non-coding (lnc) RNA and mRNA associated with oxaliplatin resistance, the authors found that lncRNA prion protein testis specific (PRNT) is upregulated in colon cancer [72]. By performing in vitro and in vivo studies, the authors found that PRNT upregulation induces colon cancer cell migration, resistance to OXA and tumor growth [72]. Analysis of datasets showed PRNT upregulation in OXA-resistant colon cancer along with downregulation of HIPK2. Mechanistically, PRNT regulates HIPK2 mRNA expression in colon cancer by sponging ZNF184 transcription factor [72] (Table 1). These findings are in line with previous studies showing the positive role of HIPK2 in drug-induced apoptosis [29].

### 2.6. HIPK2 and NRF2

It has been recently evidenced that nuclear factor erythroid 2 (NF-E2) p45-related factor 2 (NRF2), encoded by the *NFE2L2* gene [101,102], is among the molecular pathways altered in colon cancer. NRF2 is the master regulator of oxidative stress that, in normal cells, is downregulated by proteasomal degradation through binding to Kelch-like ECH-associated protein 1 (Keap1). Under oxidative stress, NRF2 is released from its inhibitor Keap1 to activate the transcription of antioxidant and cytoprotective genes [103]. Although NRF2 is a cytoprotective factor and its transient activation is linked to chemoprevention, its sustained activation has been shown to protect cancer cells against chemo- and radiotherapy and promote metabolic activities that support cell proliferation and tumor growth [104]. For this opposite behavior, NRF2 is considered a “double face” molecule [105]. In solid cancer, NRF2 is upregulated by several mechanisms (e.g., Keap1 inhibition, *NFE2L2* gene mutation, oncogene-induced transcription of NRF2 via KRAS, BRAF, MYC activation), promoting cell proliferation, resistance to therapy and an unfavorable prognosis [106]. For this reason, NRF2 has recently drawn the attention of many researchers for its role as a biomarker of therapy response and prognostic factor in tumors. A role for NRF2 in colon cancer progression has been unveiled and associated with patients’ poor prognosis and resistance to chemo-and radiotherapy [101,107,108,109]. Mechanistically, overexpression of NRF2 in colon cancer tissues, compared to normal tissues, has been found to correlate with ERK1/2 and AKT signaling pathway activation [109]. Although with the limitation due to small samples size, NRF2 expression was related to pathologic stage, indicating that overexpression of NRF2 may be linked with formation and stage of colon cancer [109]. These findings were in agreement with the study analyzing NRF2 expression in 76 colon cancer patients’ tissues and paired normal tissues showing that NRF2 protein expression is significantly higher in cancer tissues and that it positively associates with larger tumor size, advanced TNM stages and metastasis [108]. In another study, by measuring the NRF2 activity in silico and performing its validation in patient samples, the authors showed that NRF2 pathway is upregulated in colon cancer and that such high NRF2 expression associates with patients’ poor prognosis [110]. Then, a correlation between NRF2 expression and reduced HIPK2 activity in colon cancer cells was found in pre-clinical studies showing that NRF2 activation correlates with the inhibition of phosphorylation of p53Ser46 and with the reduction of cisplatin-induced cell death, highlighting an interplay between NRF2 and HIPK2/p53 axis in response to anticancer chemotherapy that can be hijacked by cancer cells to bypass drug cytotoxicity [43,44]. In this regard, NRF2 has been suggested to engage with the HIPK2 protein in a pro-survival crosstalk to the detriment of the HIPK2 apoptotic activity [42]. In that setting, HIPK2 has been shown to induce some antioxidant target genes that it has in common with NRF2 (i.e., NQO1, HO-1), promoting cancer cell survival [42]. Therefore, NRF2 activation in colon cancer, as for instance in the above studies [101,108,109,110], may impair HIPK2/p53 apoptotic activity, and favor HIPK2-dependent transcriptional program of genes promoting chemoresistance and tumor progression. Further studies are necessary to demonstrate that hypothesis and the underlying molecular mechanisms of the NRF2/HIPK2 interplay in colon cancer, but also in other types of cancers, tilting the death/survival outcome. In this regard, targeting NRF2 in tumors might be a useful strategy to restore cancer chemosensitivity [111] and likely HIPK2 apoptotic activity. As a proof of principle, inhibition of NRF2 has been shown to selectively induce cell death in NRF2-addicted colorectal cancer cells by promoting ferroptosis and pyroptosis [112,113] and to increase chemotherapy response only when HIPK2 is expressed [71].

## 3. HIPK2 Role in the Colon Cancer Tumor Microenvironment (TME)

In the tumor–host interaction in the TME, a key role for tumor progression is played by angiogenesis, the formation of new blood capillaries taking place from preexisting functional vessels [114]. Angiogenesis contributes to tumor growth, resistance to therapies, inhibition of apoptosis, tumor invasion and metastasis and, for those reasons, is considered a hallmark of cancer progression [115]. In many solid cancers, angiogenesis is constantly activated by the “angiogenic switch” that is triggered by hypoxia, a common condition in solid cancers [116]. Hypoxia leads to the transcription of several angiogenic factors, the most important being VEGF [117], by means of the HIF-1 transcription factor [80]. HIF-1 is a heterodimeric molecule consisting of the constitutively expressed HIF-1β subunit and the hypoxia-stimulated HIF-1α subunit [118]. After dimerization with HIF-1β, HIF-1α binds to a consensus sequence called hypoxia-response element (HRE) and controls, in the nucleus, the expression of several genes involved in many aspects of cancer progression, including angiogenesis, metabolic adaptation, apoptosis resistance, invasion and metastasis [80,119]. In the extracellular milieu, important mediators of intercellular communications are the exosomes, i.e., spherical membrane-closed nanovesicles secreted by all types of cells [120]. Tumor cells produce abundant exosomes that participate in intercellular communication performing intercellular transfer of components such as microRNA (miRNA), locally and systemically interacting with the surrounding cells in the TME [121]. Growing evidence suggests that tumor-derived exosomes (TEXs) are considered new players in tumor growth and invasion, tumor-associated angiogenesis, tissue inflammation and immunologic remodeling [19]. Exosomes and their cargos may serve as cancer prognostic marker, therapeutic targets or even as anticancer drug carriers [122].

**Figure 3:**
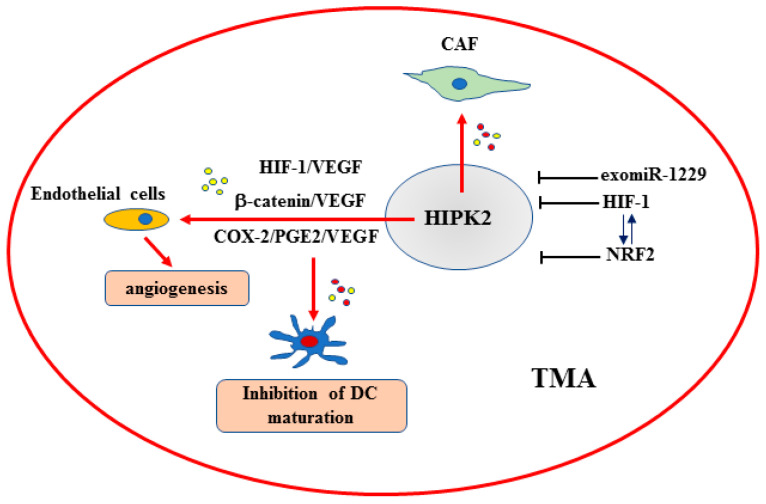
Schematic representation of the HIPK2 role in the TME (tumor microenvironment). HIPK2 can be inhibited by exomiR-1299, HIF-1 and NRF2 (blocking black lines). HIF-1 and NRF2 can sustain their oncogenic ability (↑↓). HIPK2 inhibition leads to increased (red arrow) differentiation of CAF (cancer-associated fibroblasts); to induction of angiogenesis by targeting endothelial cells with secreted (red arrow) VEGF (vascular endothelial growth factor) by HIF-1, β-catenin or COX pathways; and to block (red arrow) dendritic cell (DC) maturation by means of PGE2 (prostaglandin E2) production.

In a series of recent reports, miRNAs have been shown to bidirectionally regulate angiogenesis in colon cancer. Many miRNAs can directly act on VEGF or inhibit angiogenesis through other pathways (e.g., HIF-1α, PI3K/AKT, etc.), while some miRNAs, specifically many exosomal (exo)miRNAs, are capable of promoting colon cancer angiogenesis [123]. A study of patients with colon cancer showed high levels of circulating exomiR-1229, which correlates with tumor size, lymphatic metastasis, angiogenesis and poor survival [73]. In colon cancer tissues, HIPK2 mRNA expression was found to be significantly downregulated compared to the adjacent normal tissues and, mechanistically, exomiR-1229 was found to target the HIPK2 3’UTR. The reduction of the HIPK2 protein levels in of human umbilical vein endothelial cells (HUVECs) upregulated the downstream VEGFA, VEGFR1 and p-AKT, thereby stimulating angiogenesis [73] (Table 1). In agreement with earlier studies [37,45,62], HIPK2 reduction in colon cancer tissues, compared to the normal tissues, could be considered a potential novel prognostic marker of colon cancer progression, along with high production of exomiR-1229 [73]. Remarkably, exomiR-1229 was found to cause breast tumorigenesis by activating the Wnt/β-catenin pathway through targeting the key negative regulators of β-catenin, such as glycogen synthase kinase (GSK)-3 β, adenomatous polyposis coli (APC) and ICAT [124]. The β-catenin transcription factor is strongly involved in the early and stepwise events of the colon tumorigenesis by activating genes such as c-myc, cyclin D1 and VEGF [125]. Aberrant activation of Wnt/β-catenin signaling has been similarly linked to the progression of many other cancer types [125,126]. HIPK2 has been shown to phosphorylate and degrade β-catenin protein, therefore repressing the Wnt/β-catenin-mediated transactivation of target genes such as cyclin D1 [38]. The knockdown of endogenous HIPK2 in colon cancer cells augments the stability of β-catenin and the expression of β-catenin target genes, stimulating proliferation, increased wound healing and in vivo tumor growth [38]. These results are in agreement with earlier results showing that HIPK2 downregulates β-catenin levels through proteasomal degradation system, an effect dependent on HIPK2 catalytic activity and independent of p53 and GSK-3β activities [36,37]. Collectively, the above findings make it tempting to speculate that high levels of exomiR-1229 might induce tumor angiogenesis not only by blocking the HIPK2-mediated suppression of VEGF expression [73] but also by blocking the HIPK2-mediated inhibition of the β-catenin/VEGF pathway (Figure 3).

In addition to β-catenin, another key transcription factor for VEGF expression is HIF-1 [118]. In vitro studies with colon cancer cells showed that HIPK2 binds, along with HDAC1, to the HIF-1α gene promoter repressing the HIF-1-mediated transcription of VEGF [85], in line with data showing that the HIPK2/HDAC1 complex leads to histone deacetylation and control of gene transcription [45,127]. As a proof-of-principle of the HIPK2/HIIF-1/VEGF regulation, conditioned media from HIPK2 knockdown colon cancer cells has been shown to enhance in vitro tube formation of HUVEC [85]. In agreement, tumor xenografts established from colon cancer cells depleted of HIPK2 function, compared to control ones, showed that HIPK2 knockdown increases tumor vascularity and blood density, as evaluated by immunohistochemical analysis and by HIF-1α and VEGF up-regulation at the mRNA levels [85]. These findings are in agreement with a study showing that HIPK2 induces HIF-1α proteasomal degradation and subsequent angiogenesis in liver cancer [128]. HIF-1α is mostly regulated at posttranscriptional levels by low oxygen conditions [129]; however, the regulation by HIPK2 at the transcriptional level [85] suggests that inhibition of HIPK2 can induce a pseudo-hypoxic phenotype with HIF-1 activation and an “angiogenic switch”, leading to tumor progression, invasion, angiogenesis and resistance to therapies, that can be applied to colon cancer and also to other solid cancers (Figure 3). Thus, HIF-1 activation has a special impact on cancer progression because of its effect on angiogenesis [82] and targeting hypoxia in tumors is a promising therapeutic strategy [130]. In this regard, our previous studies showed that zinc supplementation may downregulate HIF-1α and revert the hypoxia-induced changes of gene expression in colon cancer cells restoring HIPK2 and p53 activities [131,132,133]. Interestingly, hypoxia has been shown to downregulate HIPK2 through Siah-2-dependent proteasomal degradation, allowing the induction of a substantial fraction of hypoxia-induced genes [48], therefore, controlling tumor progression and response to therapies. To make the interplay even more complicated, an intriguing link between HIF-1 and NRF2 has been highlighted [134]. In xenograft models of tumors formed by colon cancer cells depleted of NRF2 function, tumors were smaller and developed fewer blood vessels, compared to control tumors, depending on the reduced expression of HIF-1α [135]. NRF2-inhibited cancer cells failed to accumulate HIF-1α protein in hypoxic conditions, likely due to reduced mitochondrial O2 consumption that enables PHD hydroxylation of HIF-1α and consequent protein degradation: this mechanism limits the expression of VEGF and other HIF-1 target genes [135]. These findings underscore an integrated interplay between HIF-1 and NRF2 in the cellular response to hypoxia and in tumor progression, highlighting NRF2 as a candidate molecular target to control angiogenesis by imposing a blockade of HIF-1 signaling (Figure 3). In addition, given the interplay between HIPK2 with HIF-1α [85,136], HIPK2 and NRF2 [42,44], and NRF2 and HIF-1, it can be speculated that HIPK2 apoptotic activity might be restrained by the HIF-1/NRF2 interplay; therefore, targeting either HIF-1- or NRF2-induced pathways or both is a possible strategy to control tumor progression, in part through reestablishing HIPK2 oncosuppressor activities.

In the TME, CAFs sustain cancer growth and support malignancy and tumor resistance to therapies in a crosstalk with cancer cells [137]. It was shown that HIPK2-modulated pathways derived from colon cancer cells depleted of HIPK2 function are involved in fibroblast transdifferentiation CAF-like [47]. Mechanistically, conditioned media of HIPK2-silenced colon cancer cells induced autophagy in fibroblasts that reduced caveolin-1 levels [47], a hallmark of the aggressive CAF phenotype in cancer patients [138]. Therefore, HIPK2 inhibition in cancer cells can also contribute to tumor progression and resistance to therapies through TME remodeling activities (Figure 3).

## 4. Conclusions

Although marked advances in colon cancer investigations have been made in the last years, colon cancer still remains the second leading cause of cancer-related mortality worldwide. Thus, additional diagnostic parameters and molecular determinants of clinical outcome and response to therapies are still required to help pave the way for the development of novel diagnostic and therapeutic strategies. This review provides information, mostly with pre-clinical evidence, about a potential candidate biomarker (HIPK2), that might aid in improving the diagnosis and help extend the treatment options of colon cancer cases, thus ameliorating the prognosis of colon cancer patients. 

HIPK2 plays a key role in cancer biology thanks to the interaction with molecular pathways involved in cancer progression and response to therapies. Regarding colon cancer, several studies have demonstrated in vitro and in tumor xenografts that HIPK2 inhibition: (*i*) increases tumor progression and reduces tumor response to therapies through inactivation of the p53 apoptotic activity; (*ii*) induces VEGF production and angiogenesis by means of HIF-1, β-catenin or COX-2 activation; (*iii*) induces a pro-inflammatory phenotype that favors tumor progression and immune evasion; and (*iv*) induces CAF differentiation in the tumor–host interaction. Intriguingly, HIPK2 can be the target of miRNAs or hypoxia that inhibit its expression and increase tumor progression or can interact with NRF2 that impairs its apoptotic activity (Figure 3). The analyses of tissue samples or genomic data sets from colon cancer patients mostly agree with the molecular outcome, that is, that high HIPK2 expression correlates with low Dukes stages and with high patient survival, suggesting that the HIPK2 presence is important in restraining tumor progression. In addition, in few studies, high HIPK2 expression in patient samples has been found to correlate with better response of, for instance, stage II colon cancer to OXA and 5-FU, and also to a better prognosis for the patients, in agreement with the HIPK2 oncosuppressor role. In only one study, high HIPK2 expression in colon cancer tissues was found to correlate with tumor progression and TNM stages, although the molecular mechanisms were not completely unveiled. In summary, the collected findings indicate that HIPK2 expression could be a useful biomarker to evaluate colon cancer progression and response to therapies, also along with the molecule pathways that interact with it, as summarized above. The findings also suggest that it is worth further studying the mechanisms that control HIPK2 protein regulation or mRNA expression and dictate the pro-survival/apoptotic balance. In addition, more in vivo experiments and analyses of large patient groups, also according to tumor stage and response to therapies, will help to better decipher the prognostic role of HIPK2 and the translational potential in clinical practice.
